# Shrinkage-Considered Mold Design for Improvement of Micro/Nano-Structured Optical Element Performance

**DOI:** 10.3390/mi11100941

**Published:** 2020-10-17

**Authors:** Minsu Kim, Eun Song Oh, Moon Kyu Kwak

**Affiliations:** 1 Department of Mechanical Engineering, Kyungpook National University, Daegu 41566, Korea; kms1685@knu.ac.kr; 2 YNG Inc., Pyeongtaek 17708, Korea; esoh@yngoptics.com

**Keywords:** micro-lens array (MLA), nano-imprint lithography (NIL), shrinkage, wafer-level optics (WLO)

## Abstract

Polymer shrinkage in nano-imprint lithography (NIL) is one of the critical issues that must be considered in order to produce a quality product. Especially, this condition should be considered during the manufacture of optical elements, because micro/nano-structured optical elements should be controlled to fit the desired shape in order to achieve the intended optical performance. In this paper, during NIL, we characterized the shrinkage of polymeric resin on micro lens array (MLA), which is one of the representative micro/nano-structured optical elements. The curvature shape and optical performance of MLA were measured to check the shrinkage tendency during the process. The master mold of MLA was generated by the two-photon polymerization (2PP) additive manufacturing method, and the tested samples were replicated from the master mold with NIL. Several types of resin were adjusted to prepare the specimens, and the shrinkage effects in each case were compared. The shrinkage showed different trends based on the NIL materials and MLA shapes. These characterizations can be applied to compensate for the MLA design, and the desired performance of MLA products can be achieved with a corrected master mold.

## 1. Introduction

Nano-imprint lithography (NIL) is an advanced lithographic technology for micro/nano devices [1,2,3,4,5]. NIL is well known for its applicable adjusted flexibility in MEMs electronics, biological applications, and polymer patterning [6,7,8,9,10,11,12]. The NIL process can achieve resolutions beyond the limits of light diffraction or scattering because it induces direct contact with the resistance for mechanical deformation of the material. Numerous technologies, such as roll-to-roll NIL, are considered simple and useful for mass production; thus, not only academia but also industries are attempting to actively apply NIL [13,14,15,16,17]. Although it can be applied to replicate fine patterns, NIL presents challenges, such as master durability, defects by contamination, large fabrication area, and shrinkage, that must be solved for its robust use. Thermosetting polymers and photopolymers commonly used in NIL processes are irreversibly hardened from their viscous resin state by heat or ultraviolet radiation. During the curing, monomer molecules are converted into a cross-linked polymer network, replacing van der Waals spaces between monomers with smaller covalent bond spaces. Resistance to polymerization shrinkage due to cross-linking by thermal or ultraviolet (UV) curing needs to be considered for precise manufacturing [18,19]. Existing studies on NIL resistance to shrinkage showed that shrinkage can reach up to 20% depending on the mold pattern size [20,21]. Such an amount of shrinkage affects the final performance of applications such as optical elements.

Micro-lens array (MLA) is one of the most important optical components in various applications, such as data storage [22], optical communication [23], imaging [24,25], illumination [26,27], and display systems [28]. In comparison with other optical elements, such as diffractive optical elements, MLA shows higher efficiency and reliability for challenging applications, such as laser homogenization in lithography illumination systems. Each lenslet in MLA plays a role in diffusing the source light with a specific divergence angle. MLA requires a tight discrepancy between the designed and manufactured geometric parameters, such as radius of curvature (ROC), size, and aspherical coefficients, to realize the intended optical performance at the design step. During fabrication of MLA with NIL, surface shape variation should be checked and controlled to secure the design parameters in fabricated samples.

The two-photon polymerization (2PP) technique, which uses a focused femtosecond pulse laser to induce polymerization of photopolymers in small voxels, has recently become a promising additive manufacturing method [29,30,31]. The rapid development of 3D printing technology using the 2PP principle has made it possible to fabricate three-dimensional shapes with nanoscale precision [32,33]. It also made it possible to quickly and easily fabricate various types of MLA with high precision. To efficiently mass-produce the 3D-printed MLAs, attempts to utilize the NIL technology for successive replication are drawing attention. However, the replicated MLAs often have different optical performance from the initial design because of the polymerization shrinkage accumulated in each replication process.

In this paper, we characterize the amount of shrinkage based on the types of resin and NIL steps with experimental results. We also present the measurement results obtained with a scanning electron microscope, 3D profiler, and white interferometer, and the optical performance of the final MLA products was measured by using a CCD camera. In addition, we suggest the shrinkage compensation method to obtain the desired performance of MLA and show the improved optical performance of the modified MLA.

## 2. Materials and Methods

### 2.1. Fabrication of Master Mold

The designed MLA geometries were fabricated into master molds using 3D direct laser writing based on two-photon polymerization via a commercial 2PP 3D printing system (Photonic Professional GT, Nanoscribe GmbH, Eggenstein-Leopoldshafen, Germany). As a substrate, fused silica glass was cleaned with acetone, isopropyl alcohol, and deionized water, followed by oxygen plasma treatment for enhanced adhesion with photocurable resin. Negative-type photocurable resin (IP-Dip, Nanoscribe GmbH) was applied onto the prepared substrate, which was then cured by femtosecond pulse laser (780 nm center wavelength, 120 mW average laser power, 100 fs pulse length, and 80 MHz repetition rate) through an objective lens (63×, NA 1.4). The fabricated master mold was passivated with C_4_F_8_ gas for low surface energy to ensure the defect-free release in the following replication process.

### 2.2. Fabrication of Working Stamp Master (WSM) and Final Product

**Figure 1:**
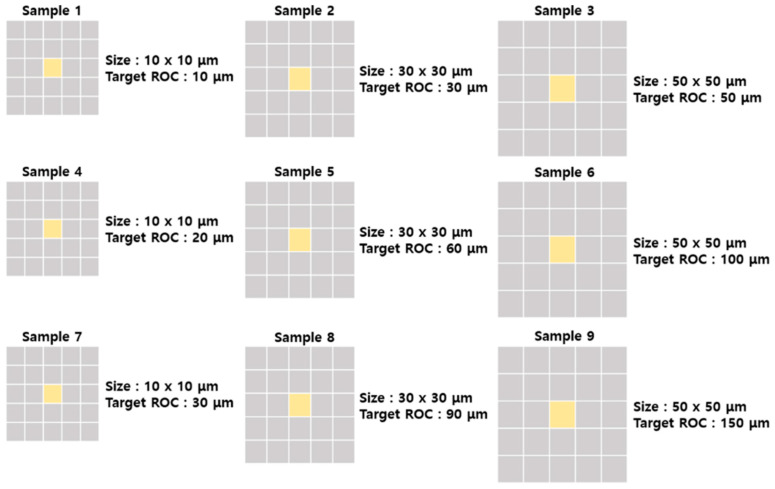
Schematic of master mold for experiments.

We designed nine types of MLA for the test and manufactured master molds. The nine types of MLA master had different three size/sags and three ROC (Figure 1). To follow the actual MLA product processing, we performed a six-step microstructure manufacturing process. The first mold replica was manufactured with polydimethylsiloxane (PDMS; Sylgard 184, Dow corning, Midland, Mich., USA) from the master mold, and the second mold replica was fabricated using polyurethane acrylate (PUA, MCnet) from the PDMS mold. Next, a WSM, as the third replica, was produced using the step-and-repeat process for wafer-level mold production, and the fourth replica was produced to obtain the final product by replicating it with PDMS or PUA on PET.

With the fourth fabricated mold replica, NIL was performed with different UV curable polymer resins (GPR-402 from MCnet, OM 625 from Delo, Ormocomp and OrmoClear FX from Kayaku). Differences were observed in the basic characteristics of each resin. In terms of shrinkage, OM 625 showed a good result, and GPR-402 was the most outstanding in terms of process convenience and stability. We measured the surface topology of samples in each step as follows: (1) master mold, (2) first replica (PDMS), (3) second replica (single mold), (4) third replica (WSM), (5) fourth replica (WSM), and (6) final sample. A total of 36 measured data for the four different polymers were compared to characterize polymer shrinkage during NIL.

### 2.3. Measurements

Scanning electron microscopy was used (S-4800, Hitachi, Tokyo, Japan) to characterize the fabricated MLA, with the operating voltage of 10–25 kV after sputtering a thin Au film (<5 nm) to avoid electron charging if necessary. Sagging depths of the micro lens were measured by a 3D profiler (Keyence VK-250K), and the curvatures of single micro lens were observed by a white interferometer (NV-3200 Nano).

## 3. Results and Discussion

### 3.1. Size Reduction by Shrinkage

**Figure 2:**
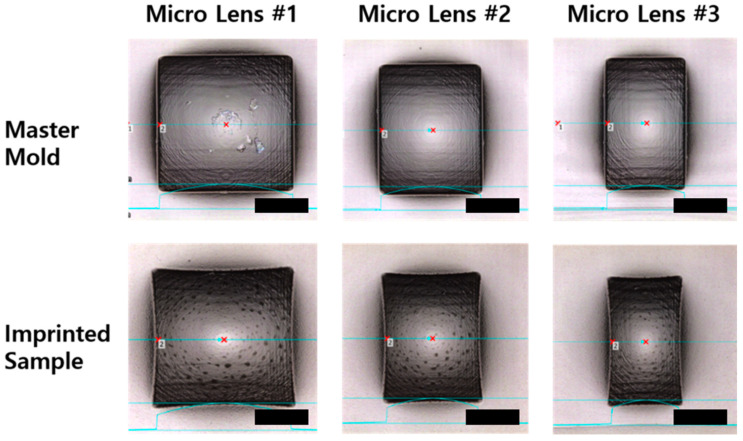
Measurement images of the master mold and imprinted single lens obtained with a micro 3D profiler. Scale bar: 20 μm.

We measured the topologies of each sample with a micro 3D profiler to determine the shape variation. Figure 2 shows the master mold shape as the first step of NIL and the topological information of the final imprinted sample. In Table 1, the measurement results of geometric values of master molds and imprinted samples showed considerable changes in the lenslet shape in MLA. The measurement results also confirmed that all geometric values of the lens were reduced (Table 1). The most striking result was that the pitch of the single lens was reduced for all three lenses—that is, from 51 to 45, 40 to 35, and 30 to 25.5 µm, in the horizontal direction. Although this measurement result proves that shrinkage occurred during imprinting, we still analyzed the shrinkage phenomenon in MLA for a reliable quantitative analysis. To clarify the analysis showing that shrinkage might have occurred during the actual process, we tested four typical resins and measured the curvature and sag of the lens for each sample. A 5 × 5 lens array was used to check the case of actual samples. A 3D profiler and white interferometer were used to measure the geometric changes.

As described in the experimental section, given that the MLA production process consists of six steps (five replicates), the sample to be measured was divided into three convex and three concave-type lenses. Given the difficulty of directly comparing the convex and concave types due to the character of the measured data, each case was divided and compared. The final product of the MLA product line used in this experiment was a concave type, and production started from the convex master mold. The lens located in the middle of the array was measured with a 3D profiler to consider the interaction caused by the contraction of neighboring lenses among the lens arrays. More detailed measurement results are described in Appendix A.

**Figure 3:**
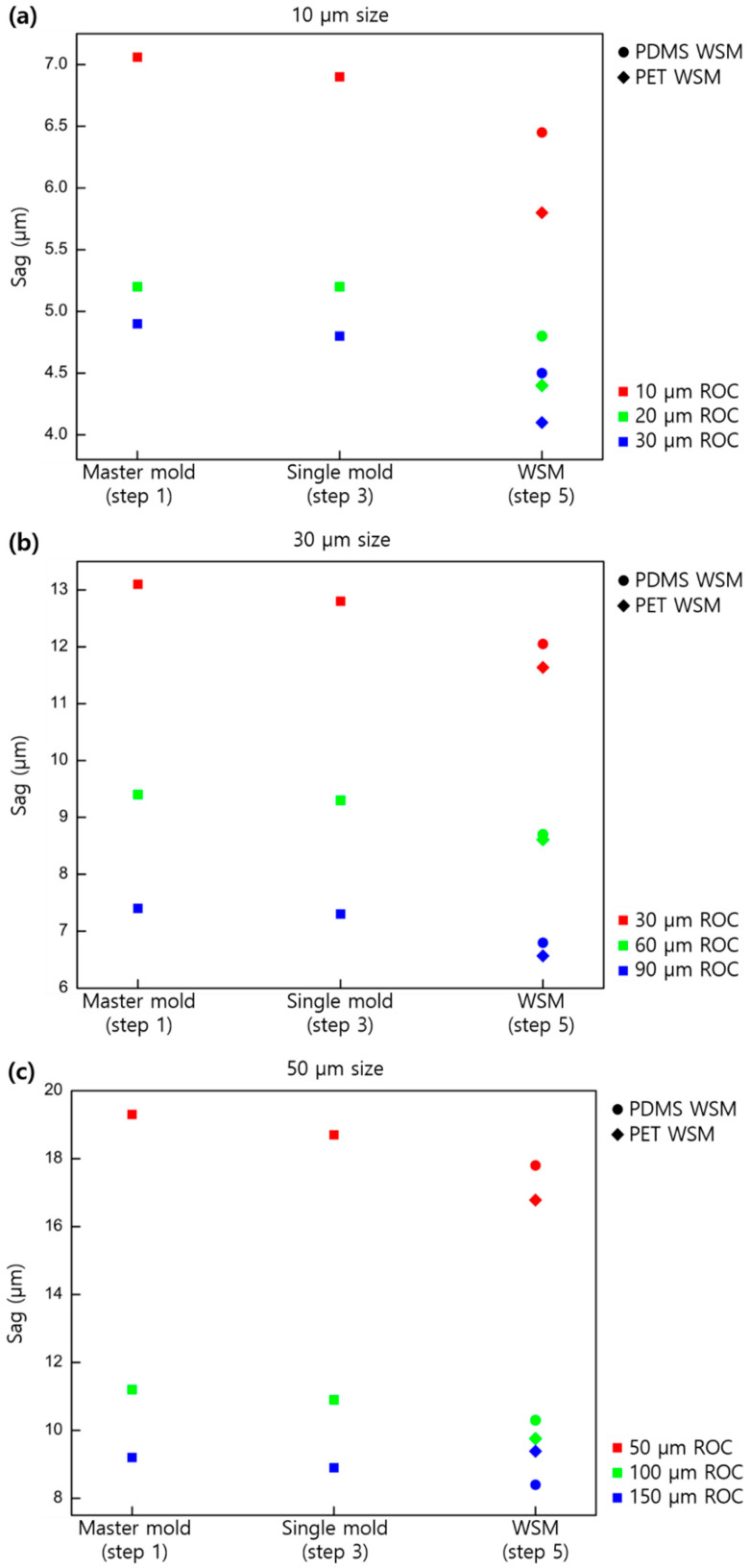
Sag profiles based on the lens size and shapes of the convex replicated samples: (**a**) 10, (**b**) 30, and (**c**) 50 µm in pitch.

Figure 3 shows the changes in the sag value for the case of a convex lens (master mold, second single-mold replica, and fourth WSM mold replica) in terms of the size and ROC of the lens.

Compared with the master mold, the second single-mold replica showed a sag reduction of around 2%, whereas the fourth replica (WSM) showed 18.3% shrinkage for the case of PUA on PET and 6.9% shrinkage for PDMS. Given the shrinkage rate, using PDMS was considered more advantageous than PUA in producing the WSM mold, which was the final mold for product production.

**Figure 4:**
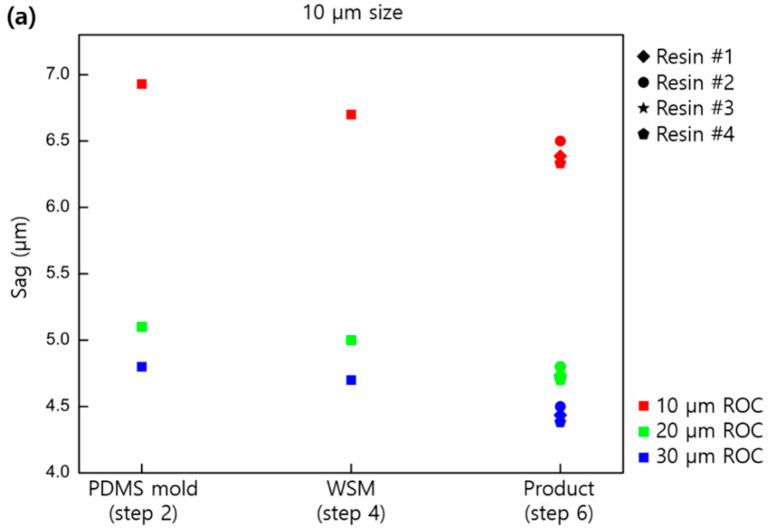

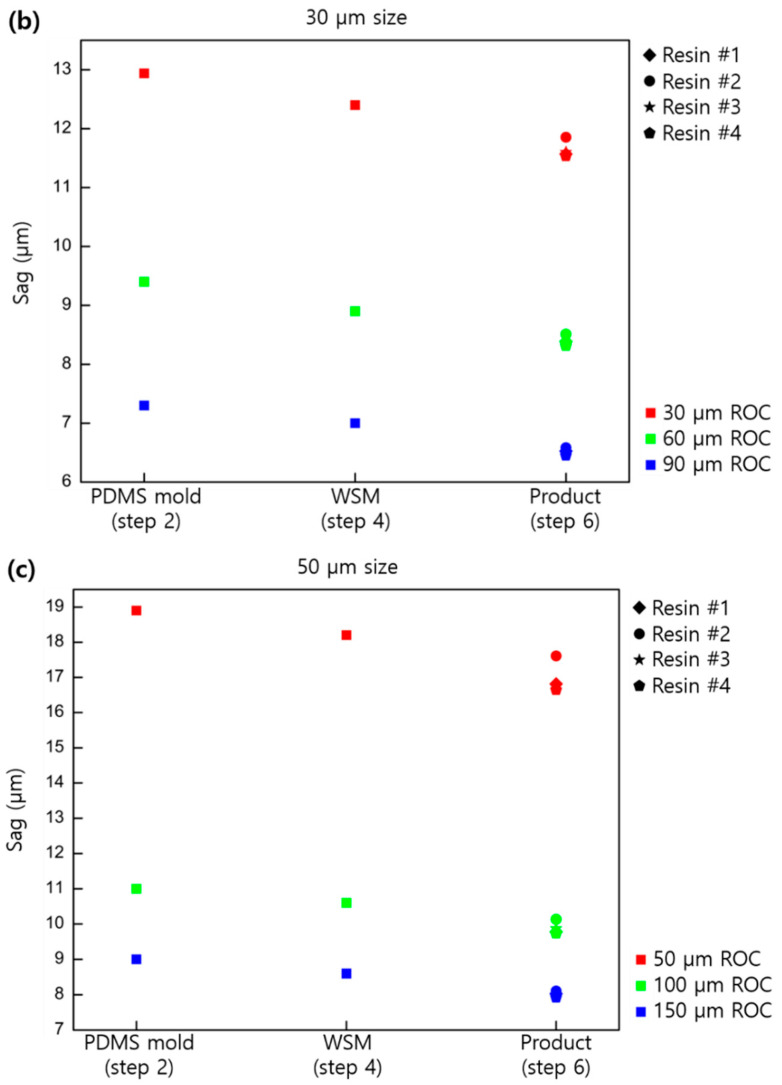
Sag profiles based on the lens size and shapes for the concave replicated samples: (**a**) 10, (**b**) 30, and (**c**) 50 µm in pitch.

Figure 4 shows the changes in sag for concave-type MLAs. As in the case of the convex type, PDMS mold (first replica), WSM (third replica), and final product were measured based on the size and curvature of the lens, and a trend similar to that of the convex type was confirmed. As in the convex case, shrinkage accumulated as the cloning process was repeated. However, shrinkage in the preparation process of the final product from WSM differed depending on the type of resin used. Table 2 shows the resins used for the final product; all resins were cured by UV light. The shrinkage of each resin showed a tendency based on the shrinkage rate reported by the manufacturer. The amount of shrinkage for each resin was constant as a result of several repeated tests.

In this process, the material used in the rest of the processes, except for the last two processes (WSM mold and final product), was not changed because the process must be established by using a material with a suitable surface energy instead of controlling the shrinkage rate. Still, the material used in all production processes should have a low shrinkage to minimize shrinkage in the final product. This condition will be studied in detail in further research.

### 3.2. Shape Change by Shrinkage

The change in the ROC of the lens was measured for nine samples of different sizes and curvatures, similar to the method of sag measurement. The ROC was measured using a white interferometer, and its value was calculated through Gaussian fitting based on the inflection point after raw data extraction. Detailed measurement results of ROC are described in Appendix A (Figure A1, Figure A2, Figure A3, Figure A4, Figure A5, Figure A6, Figure A7, Figure A8, Figure A9, Figure A10, Figure A11, Figure A12, Figure A13, Figure A14, Figure A15, Figure A16, Figure A17, Figure A18, Figure A19 and Figure A20).

**Figure 5:**
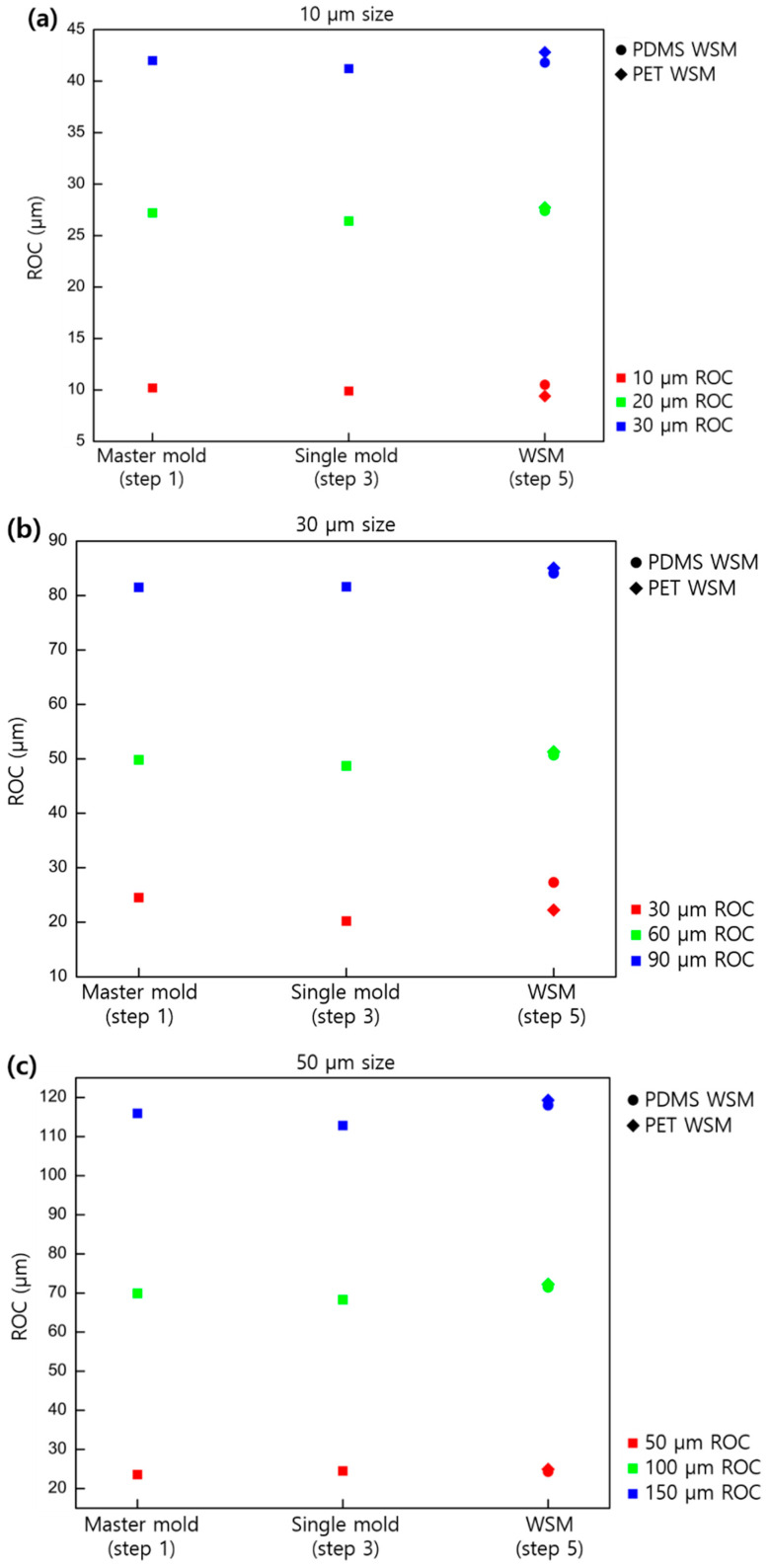
ROC of lens variances based on lens size and shapes for the convex replicated samples: (**a**) 10, (**b**) 30, and (**c**) 50 µm in pitch.

ROCs were also measured and compared for the convex- and concave-type lens separately. Figure 5 shows the ROC variance in nine different lens samples in three processing steps: master mold, second single-mold replica, and fourth replica (WSM).

Given the measurement and fitting error of the white interferometer, the experimental data values in Figure 5 were expected to have an error of around ±2.5%. With this aspect, the variance of ROC of the convex-type MLA at each stage is insignificant. The shape of the lens showed no change, unlike the previously observed decrease in sag. Thus, in the case of convex-type MLA, the shape of the lens was maintained as the resin contracted isotropically.

**Figure 6:**
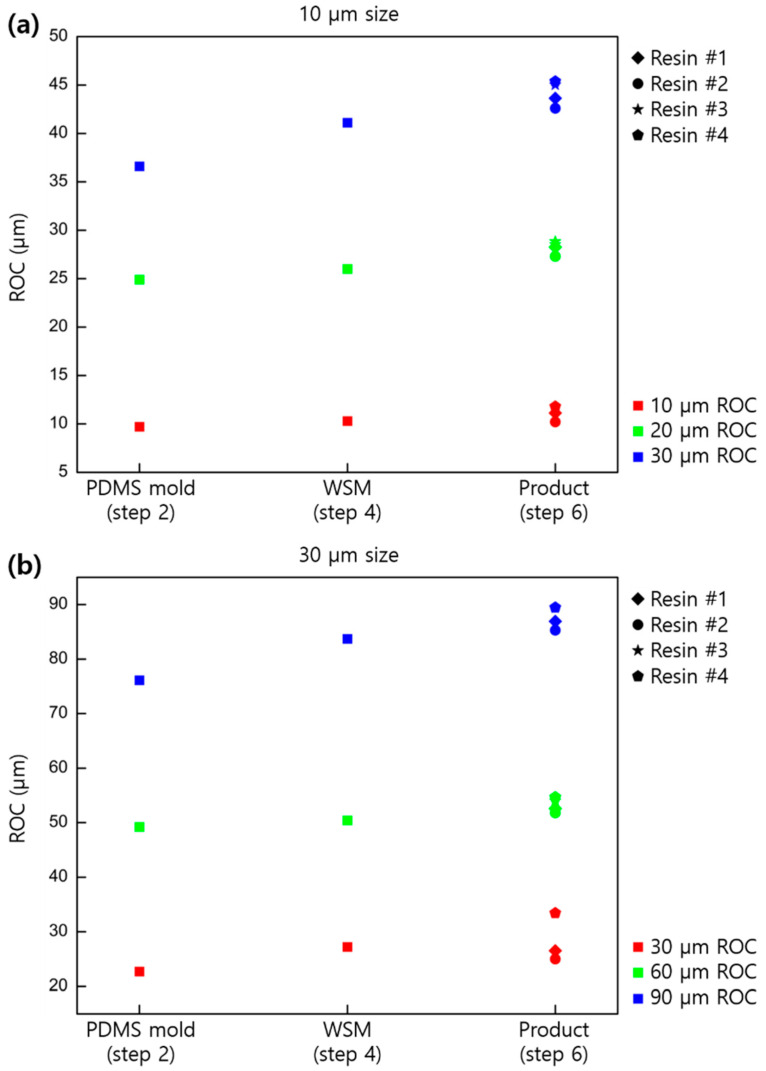

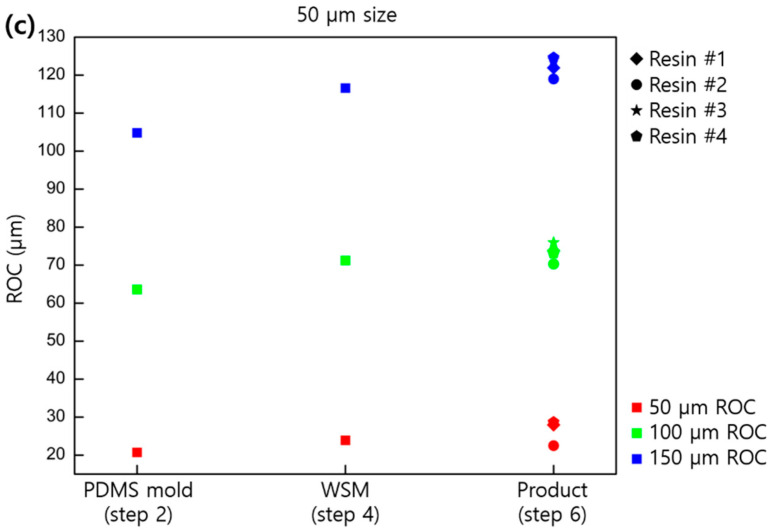
ROC of lens variances based on lens size and shapes for the concave replicated samples: (**a**) 10, (**b**) 30, (**c**) and 50 µm in pitch.

On the other hand, the concave-type lens showed a slightly different tendency (Figure 6). In the first mold replica (PDMS), third replica (WSM), and the final product, the change in ROC was measured. Thus, the tendency of the radius to increase as replication continued can be confirmed. In particular, the change in radius at the last product stage was large, and this change was considered the effect of flattening the lens as the sag of the lens changed due to contraction rather than the change in the curvature itself. Therefore, vertical shrinkage is more inclined to change the shape of MLA than other directional shrinkage during replication.

### 3.3. Total Shrinkage

**Figure 7:**
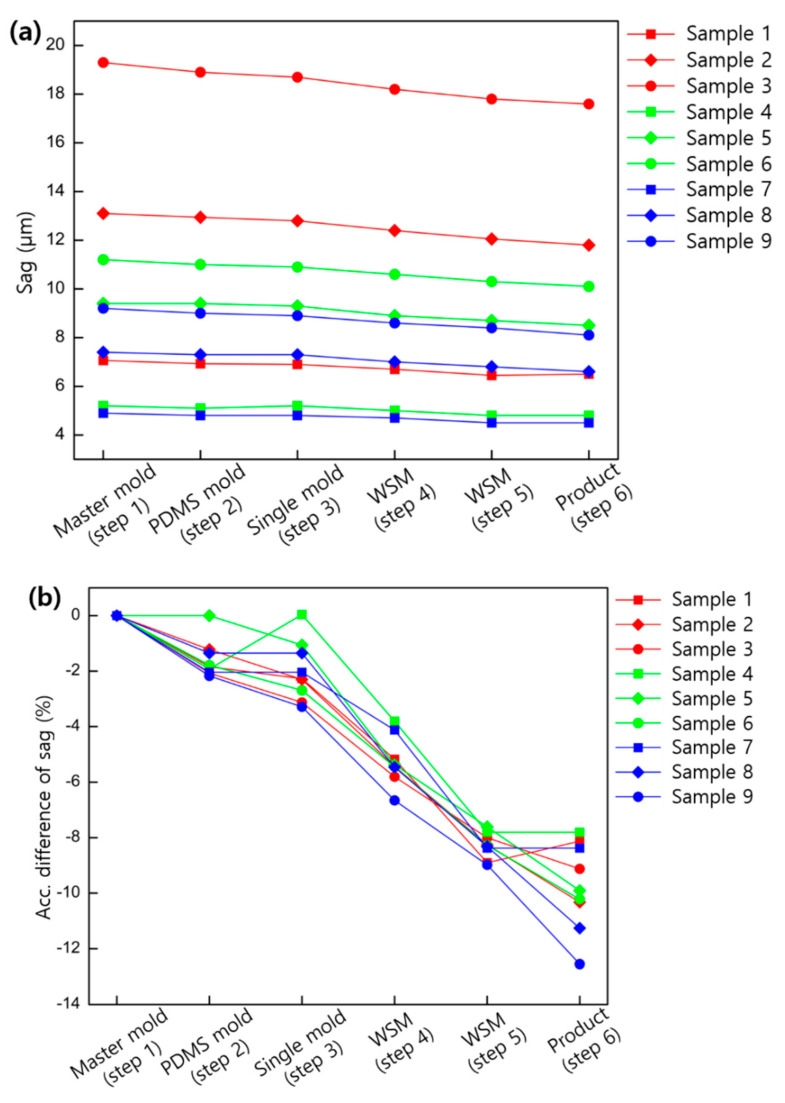

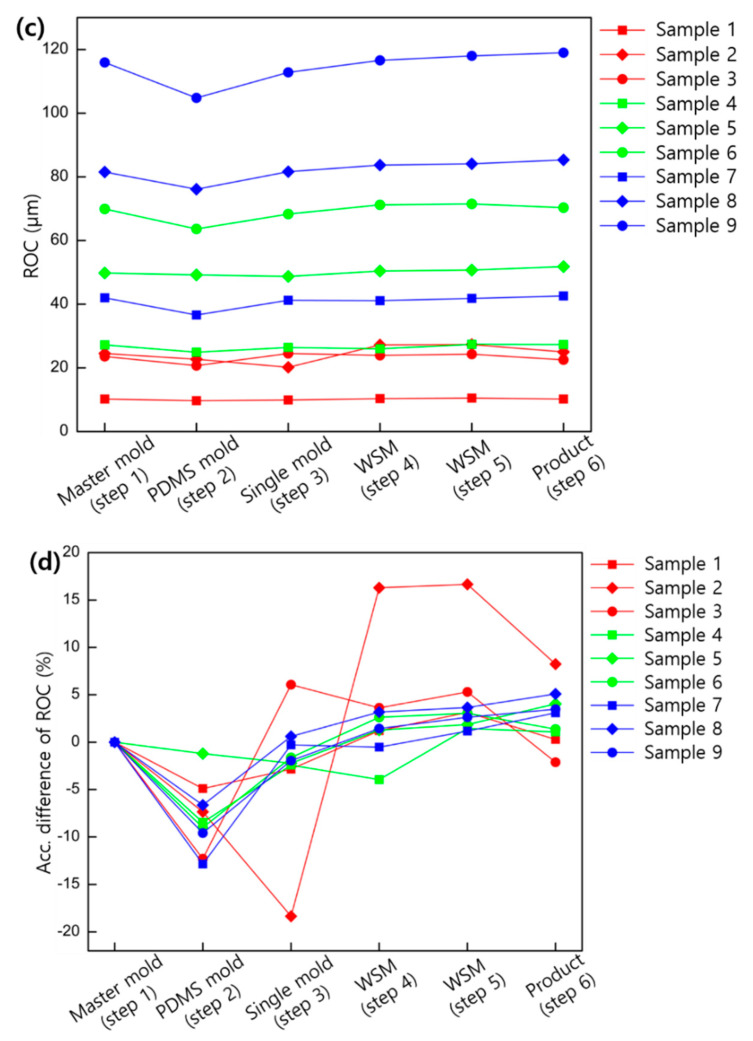
(**a**) Sag profiles of every sample at each replication step from the master mold to the final product. (**b**) The accumulated difference in sag in percent at each replication step. (**c**) ROC of lens variances at each replication step. (**d**) The accumulated difference of ROC in percent at each replication step.

The measured values of sag and ROC for each step of the entire replication process are shown in Figure 7 in the case of using PDMS and OM 625, which showed the least shrinkage in replication step 5 and step 6, respectively. The effect of polymerization shrinkage on the lens shape in the entire replication process from the master mold to the final product is shown in Figure 7. The sag of the final product shrank 8.1% at least and 12.6% at most compared to the master mold, and the larger the size and ROC of the lens, the more shrinkage tends to occur. The ROC of the final product increased at least −2.1% and up to 8.2% compared to the master mold, and the larger the lens size and ROC, the more the curvature tends to decrease.

### 3.4. Optical Performance with Shrinkage Compensation

**Figure 8:**
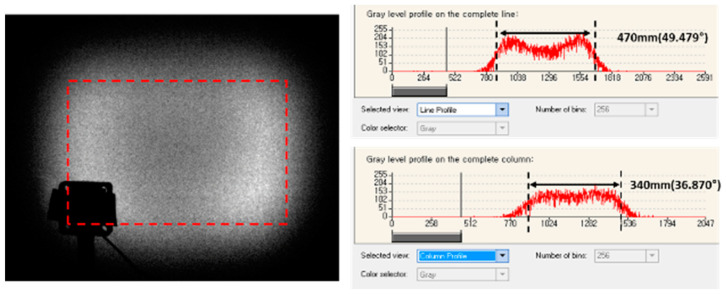
FOV measurement results of the final product sample before compensating for the shrinkage effects.

When making a light diffuser using MLA, the shape of the single lenses plays the most important role in determining the field of view (FOV) of the diffuser. The MLA for diffusing to 60° × 45° FOV was selected as a reference test. First, when the final MLA product was measured, a smaller FOV angle was measured compared with the expected value at the design stage (Figure 8). The simulation result on the designed MLA was expected to have 60° × 45° FOV, but measurement in the final NIL sample showed 49.5° × 36.9° FOV. The discrepancy possibly occurred due to polymer shrinkage effects during the NIL process. The results show that almost 21% shrinkage occurred on the lenslet surface. All the shrinkages in the five-step replication process were superimposed. Thus, such an amount of shrinkage can occur in the actual NIL process.

**Figure 9:**
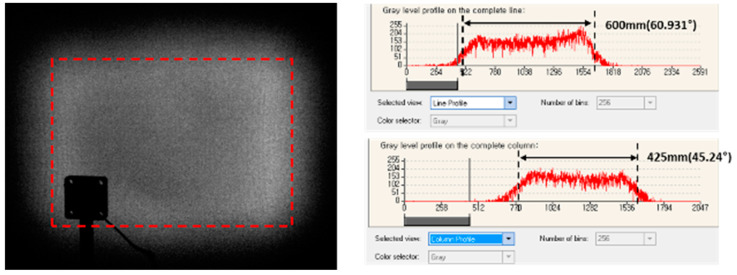
FOV measurement results of the final product sample after compensating for the shrinkage effects.

To achieve the desired FOV, we considered the shrinkage effect in the design to compensate for the sag data of each lenslet in the MLA. The compensated amounts were the measured diminished sag level. Figure 8 shows the enhanced optical performance fitted to the desired FOV value. The compensated final product covered to 60.9° × 45.2° FOV. (Figure 9) The residual angles differed from those of the requirement under 1.5%.

## 4. Conclusions

In this paper, we investigated the problems caused by the shrinkage phenomenon, which may occur in the NIL process, which is considered an essential step for the mass production of MLA, a core optical device that has recently been in the spotlight in the industry. The solutions for such problems were studied. For MLA, the light source should be diffused evenly in the desired area to perform its function in the application product, and for this purpose, the design of the micro lens shape must be conducted with precision. However, the imprinting process inevitably causes shape change as much as the amount of inherent shrinkage of the resin in the duplication process, and as a result, the desired optical device performance cannot be exhibited. In this study, the characteristics of shrinkage in MLA production were studied through various experiments and measurements. The convex-type MLA contracted isotropically, but in the concave-type MLA pattern, lens sag was the main form of contraction. In addition, the amount of shrinkage depended on the basic properties of the resin used. Thus, a corrected lens was designed based on the process to be carried out. Furthermore, the desired FOV must be obtained using an appropriate correction design to manufacture the MLA product. In this study, shrinkage appeared characteristically and consistently depending on the types of structure and resin used in the NIL process; the modified design that predicted this change is sufficiently meaningful.

## Figures and Tables

**Table 1:** Measurement data for sag and size of single lenses.

	Sag (Horizontal)		Sag (Vertical)		Max. Length (Horizontal)		Max. Length (Vertical)	
Unit: µm	Master	Replica	Master	Replica	Master	Replica	Master	Replica
Micro Lens #1	28.9	26.7	28.6	26.7	12.2	11.9	16.3	15.6
Micro Lens #2	26.2	24.1	25.9	24.1	9.5	9.4	16.4	15.4
Micro Lens #3	23.9	21.7	23.9	21.7	7.1	6.9	15.8	14.6

**Table 2:** Resins used for final imprinted product.

	Material Name	Volume Shrinkage (%)	Reflective Index
Resin #1	GPR-402 ^1^	7.5–8	1.471
Resin #2	OM 625 ^2^	2.5–3	1.572
Resin #3	OrmoComp ^3^	5–7	1.520
Resin #4	OrmoClear FX ^3^	3–5	1.555

^1^ MCnet (https://mcnnet.modoo.at/) ^2^ Delo (https://www.delo-adhesives.com/) ^3^ Kayaku advanced materials (https://kayakuam.com/).

**Figure A1:**
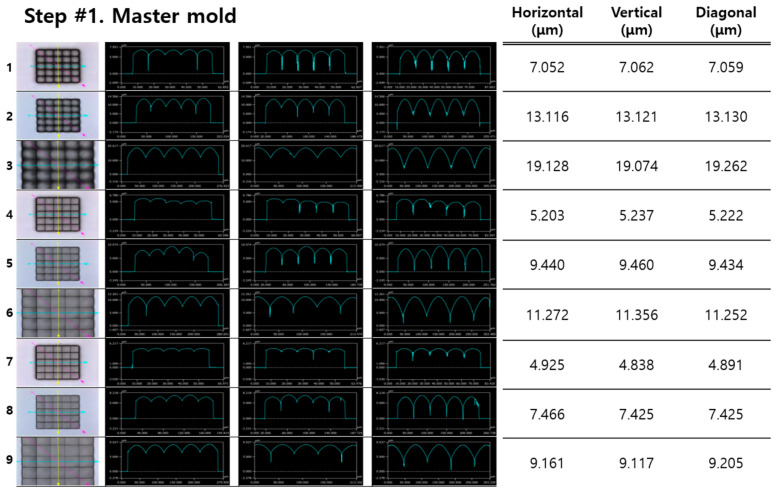
3D profiler measurement data for master mold.

**Figure A2:**
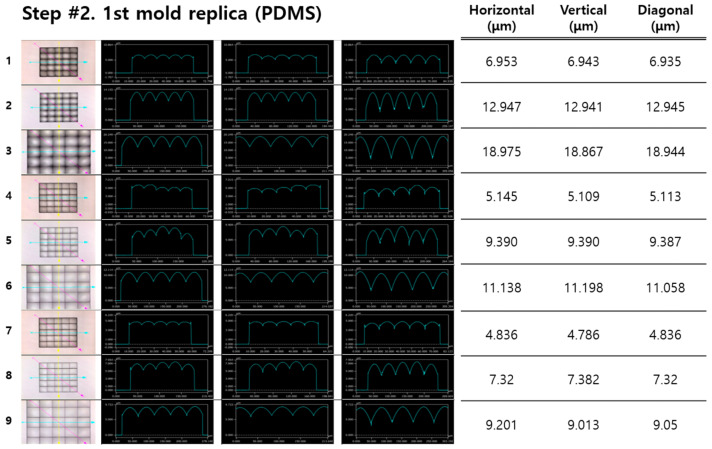
3D profiler measurement data for 1st mold replica (PDMS).

**Figure A3:**
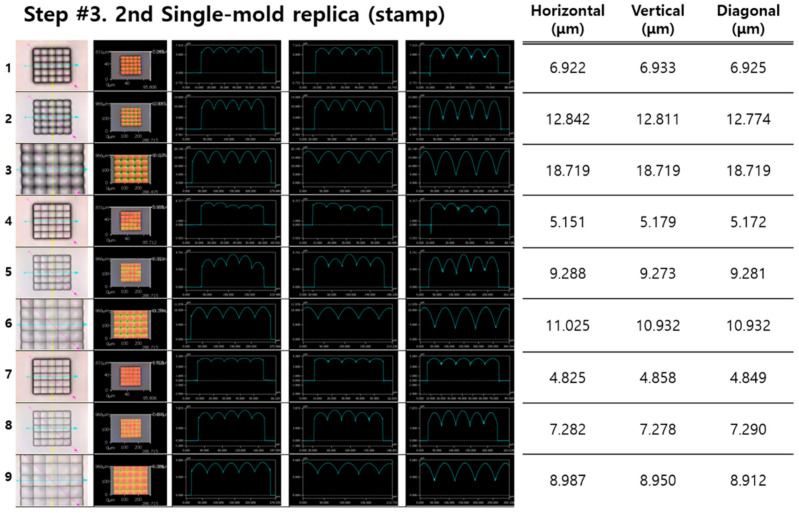
3D profiler measurement data for 2nd single-mold replica (stamp).

**Figure A4:**
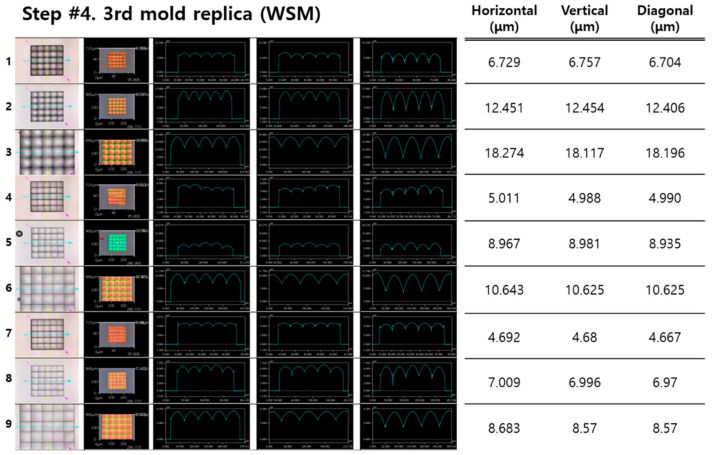
3D profiler measurement data for 3rd mold replica (WSM).

**Figure A5:**
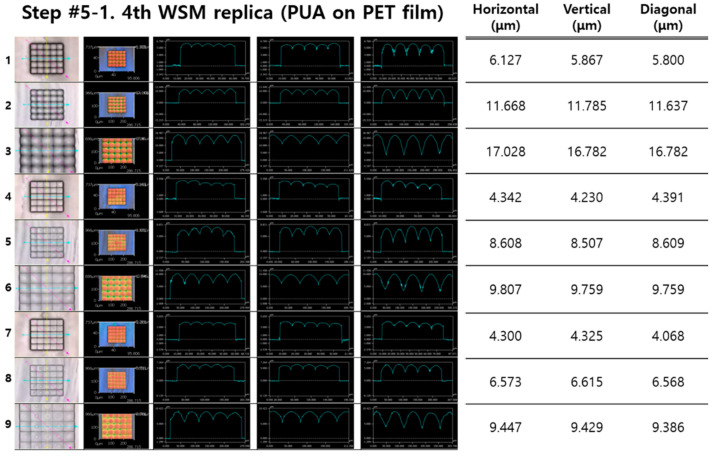
3D profiler measurement data for 4th WSM replica (PUA on PET film).

**Figure A6:**
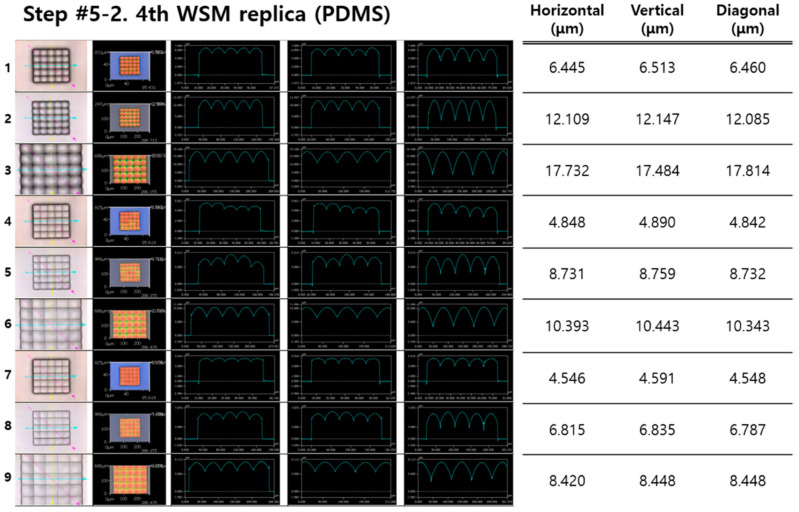
3D profiler measurement data for 4th WSM replica (PDMS).

**Figure A7:**
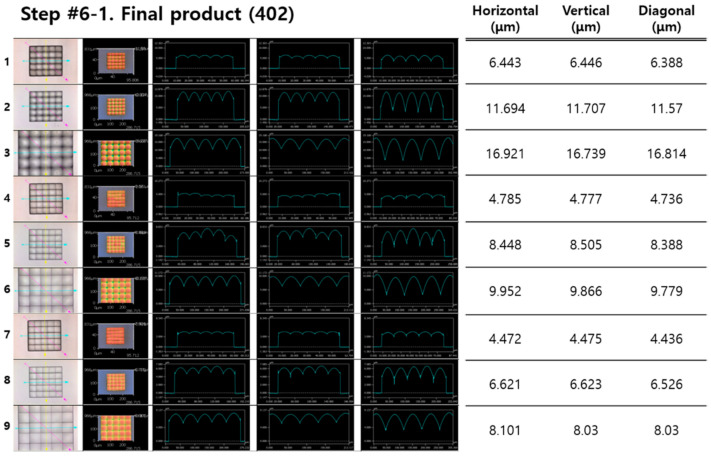
3D profiler measurement data for 5th replica of the final product (402).

**Figure A8:**
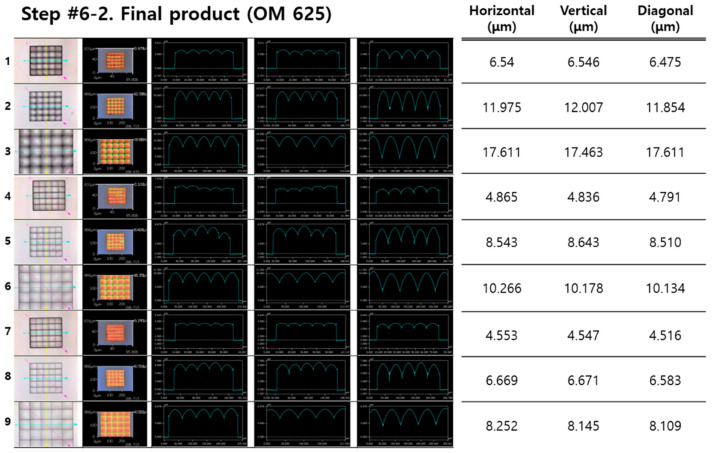
3D profiler measurement data for 5th replica of the final product (OM 625).

**Figure A9:**
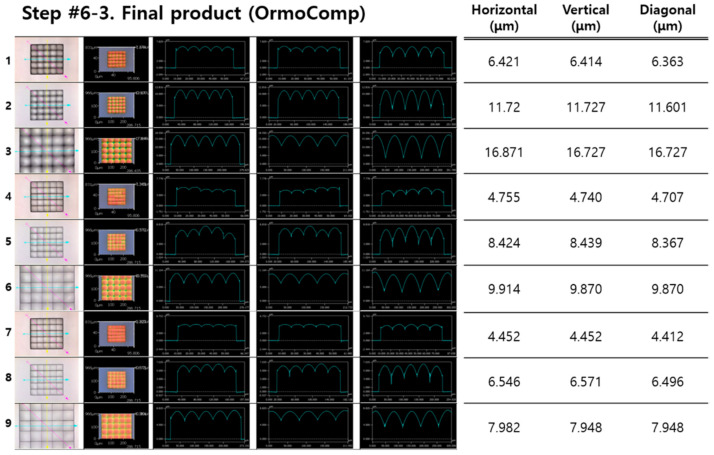
3D profiler measurement data for 5th replica of the final product (OrmoComp).

**Figure A10:**
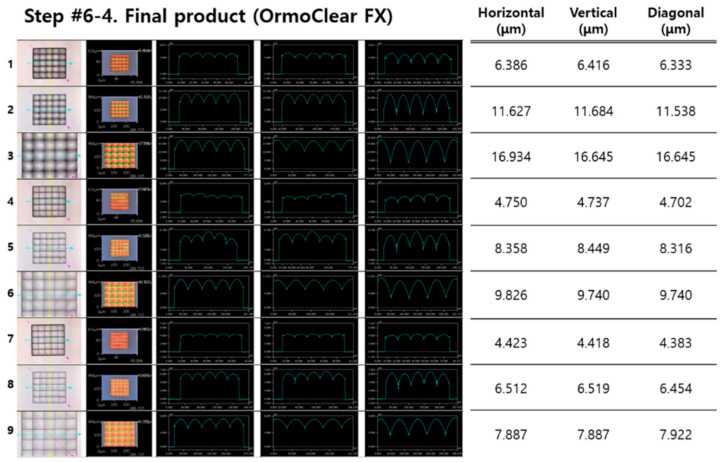
3D profiler measurement data for 5th replica of the final product (OrmoClear FX).

**Figure A11:**
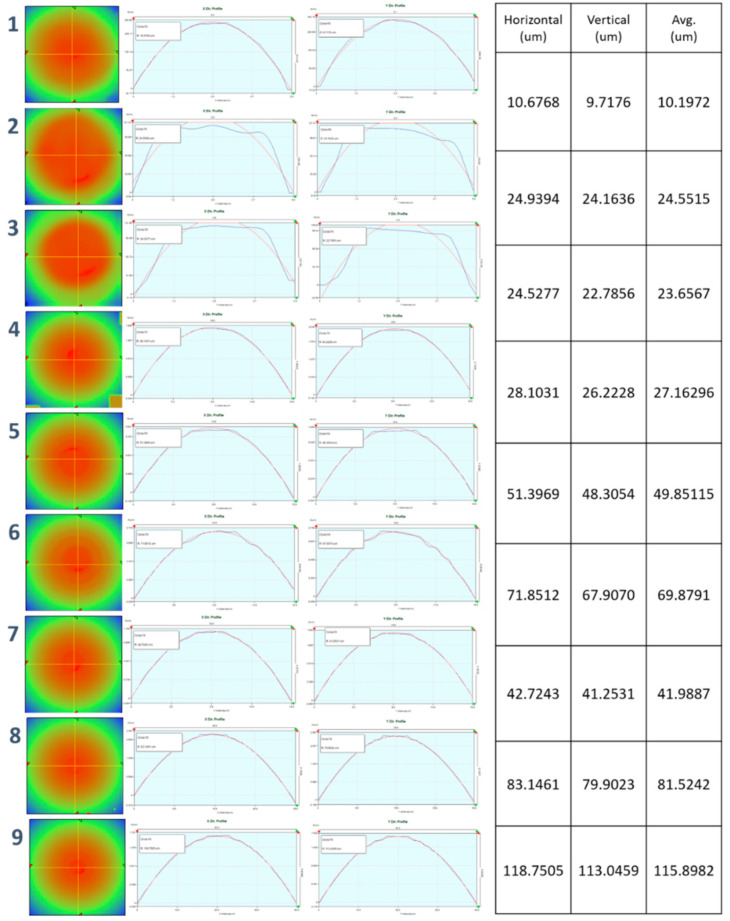
ROC measurement data for master mold.

**Figure A12:**
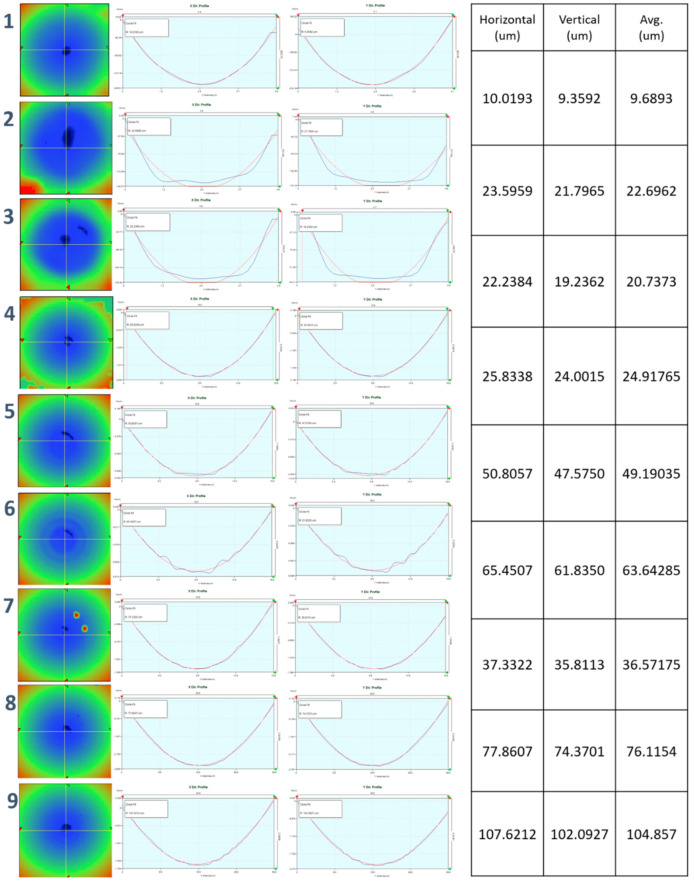
ROC measurement data for 1st mold replica (PDMS).

**Figure A13:**
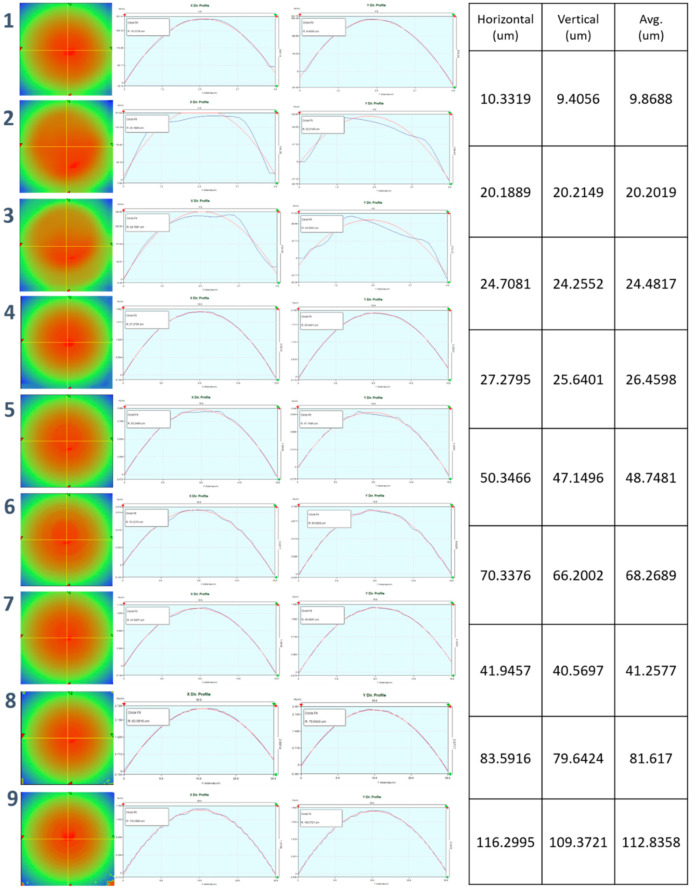
ROC measurement data for 2nd single-mold replica (stamp).

**Figure A14:**
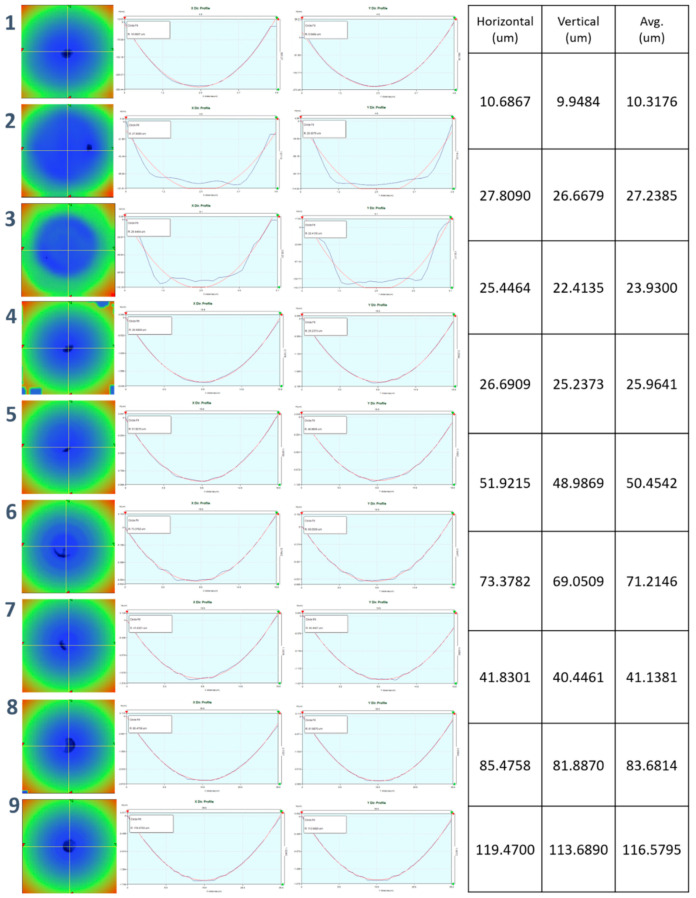
ROC measurement data for 3rd WSM replica.

**Figure A15:**
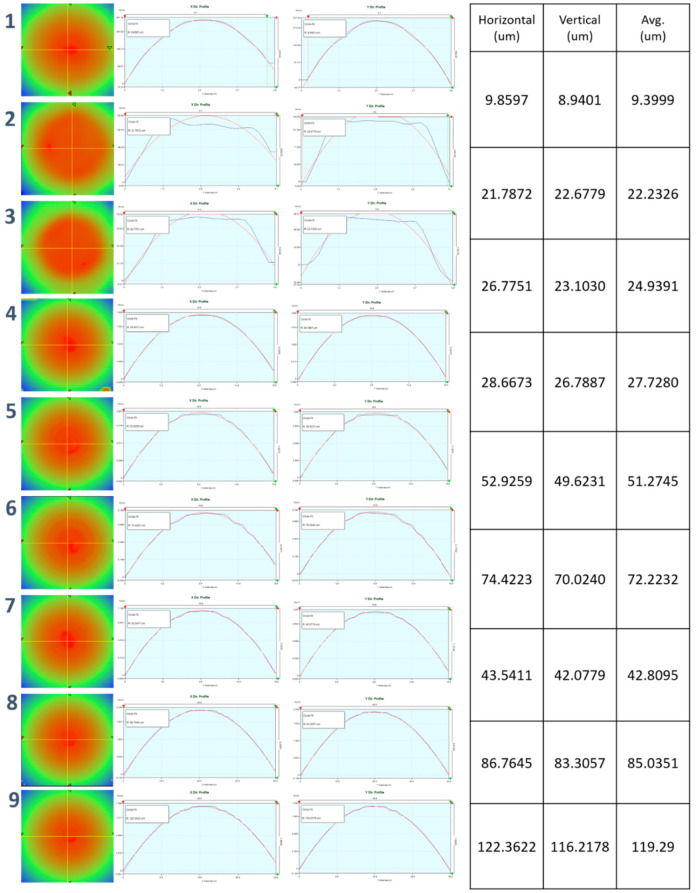
ROC measurement data for 4th WSM replica (PUA on PET film).

**Figure A16:**
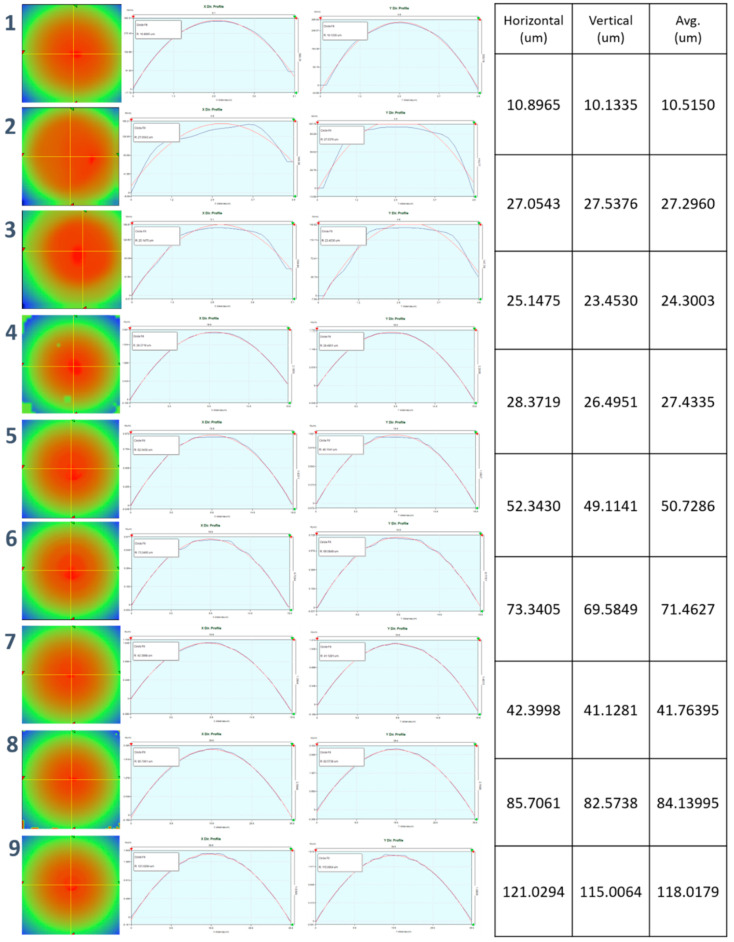
ROC measurement data for 4th WSM replica (PDMS).

**Figure A17:**
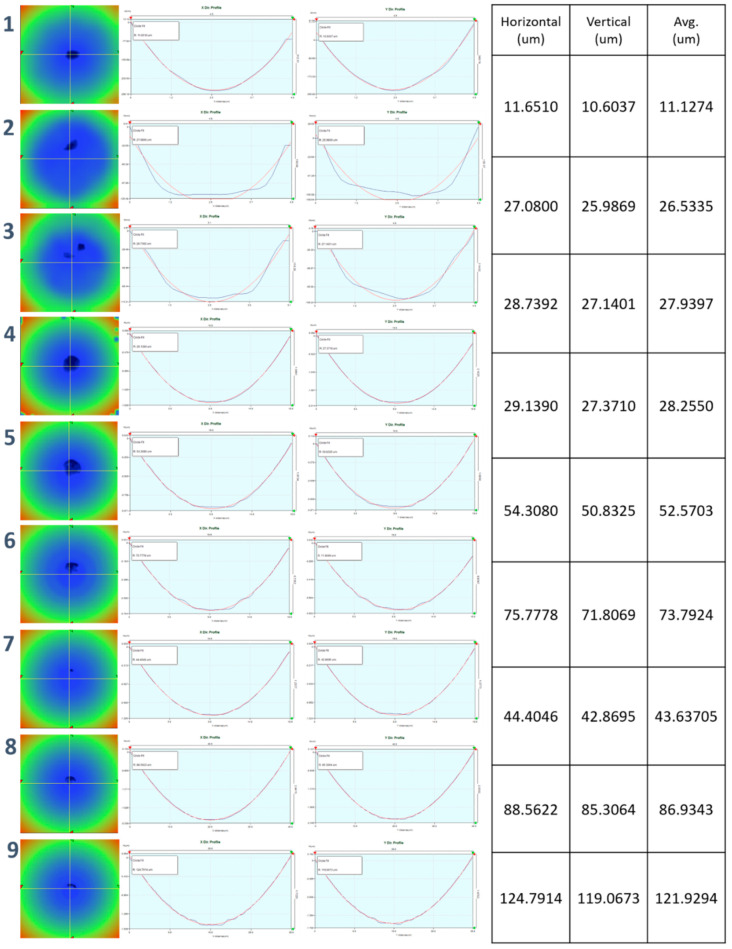
ROC measurement data for 5th final product replica (402).

**Figure A18:**
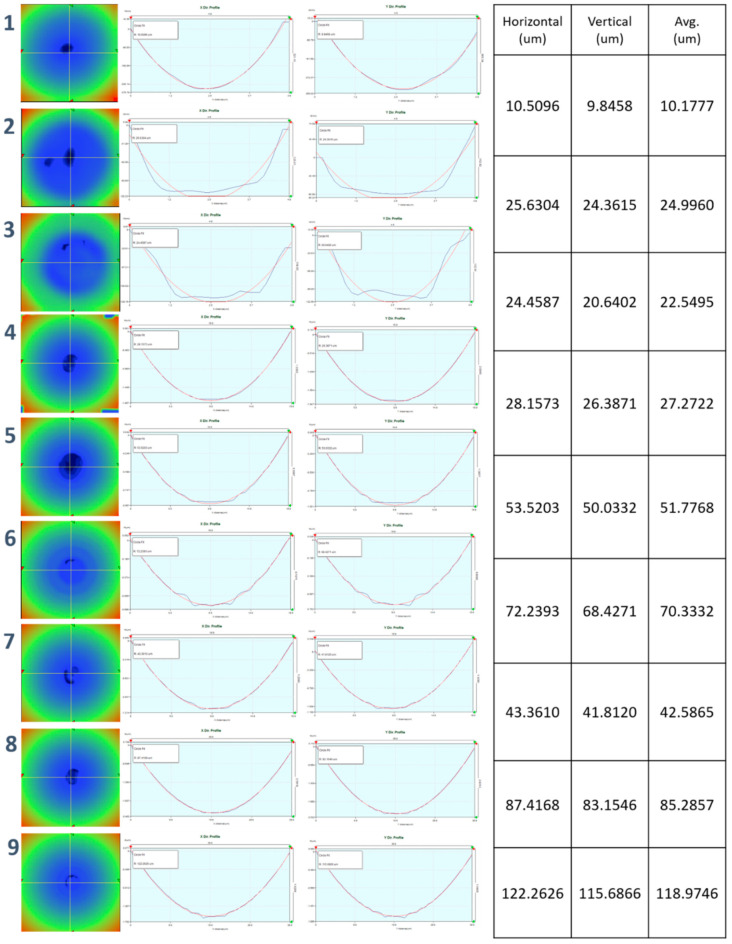
ROC measurement data for 5th final product replica (OM 625).

**Figure A19:**
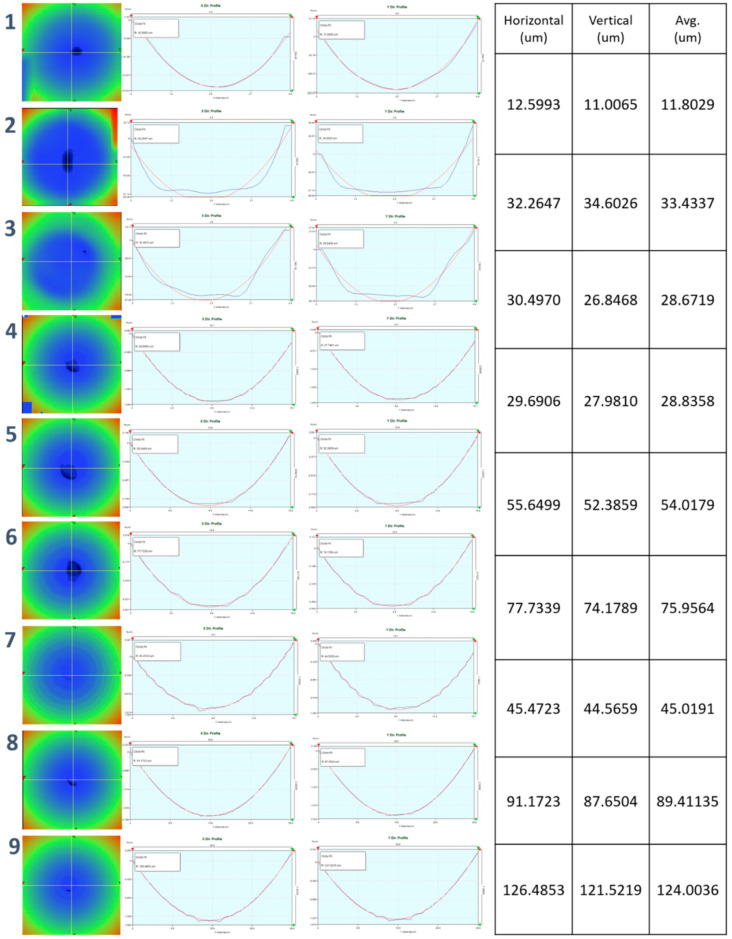
ROC measurement data for 5th final product replica (OrmoComp).

**Figure A20:**
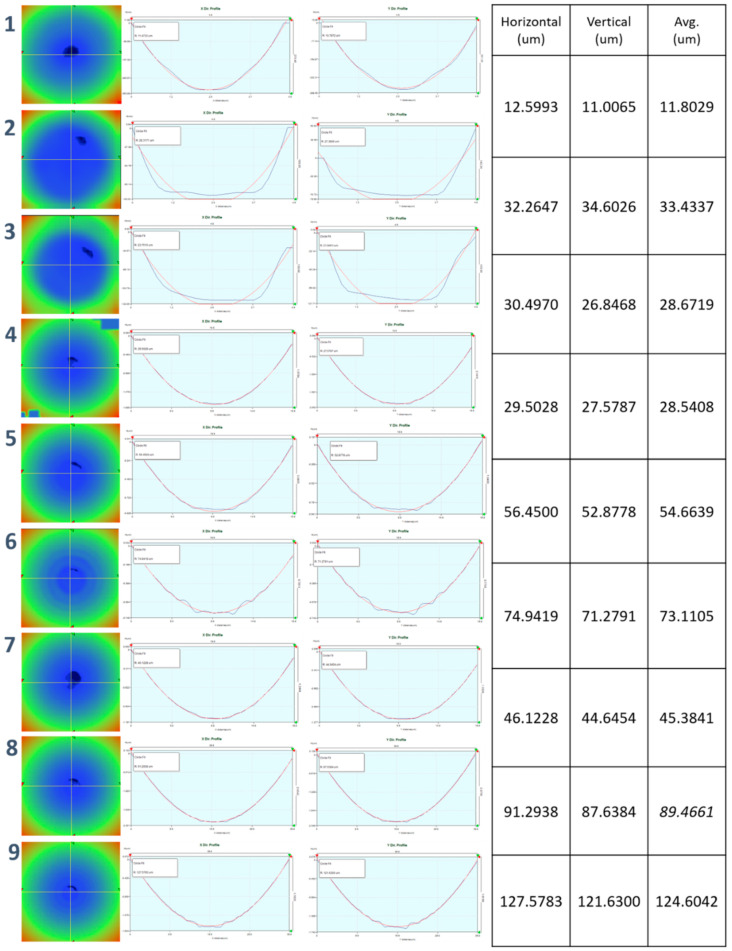
ROC measurement data for 5th final product replica (OrmoClear FX).
